# Mechanical Behavior and Predictive Modeling of Cementitious Composites Incorporating Recycled HDPE

**DOI:** 10.3390/polym18010087

**Published:** 2025-12-28

**Authors:** Omer Fatih Sancak, Muhammet Zeki Ozyurt

**Affiliations:** 1 Department of Construction Technology, Dogus University, Istanbul 34722, Turkey; osancak@dogus.edu.tr; 2 Department of Civil Engineering, Sakarya University, Sakarya 54050, Turkey

**Keywords:** HDPE, concrete, mortar, predictive modeling, mechanical properties

## Abstract

In this study, High-Density Polyethylene (HDPE) granules were used as fine aggregate replacements in concrete to contribute to plastic waste recycling. Substitution rates were determined as 10%, 20%, and 30% by volume. Slump, density, and mechanical strength tests were applied to concrete samples. All strength values decreased as the HDPE substitution rate increased. Compressive strength decreased by 12–34%, tensile strength by 7–23%, flexural strength by 6–21%, and the modulus of elasticity by 15–34%. However, axial and lateral strain values increased between 4% and 44%. Density and slump values also decreased by 3–9% and 4–19%, respectively. Additionally, a database of previously published research on concrete and mortar was compiled and integrated with the experimental results obtained. This combined dataset was used to develop predictive models assessing the influence of HDPE substitution on the mechanical performance of cementitious composites. Exponential equations were formulated to estimate compressive strength, tensile strength, flexural strength, and modulus of elasticity. These formulations were compared with existing models reported in the literature. Statistical evaluation was conducted to measure predictive accuracy, and the results demonstrated that the models proposed in this study provided superior performance relative to earlier approaches.

## 1. Introduction

High-Density Polyethylene (HDPE) is among the most widely manufactured and consumed thermoplastic polymers worldwide. It is commonly used in packaging, household products, piping systems, and construction materials. Owing to its semi-crystalline structure, HDPE demonstrates high hardness and strong chemical resistance; however, it is less flexible than other polyethylene types. Its density typically exceeds 0.941 g/cm^3^, and the molecular chain arrangement provides considerable tensile strength [1]. The favorable strength-to-density ratio makes HDPE both lightweight and durable. With a melting point in the range of 129–140 °C and an ignition temperature of about 487 °C, HDPE exhibits notable stability under elevated temperatures [2].

The rapid increase in plastic waste has become one of the most pressing environmental challenges of our time [3,4,5,6]. This growth has created significant waste management problems across multiple sectors, while the persistence of plastics in terrestrial and marine ecosystems has further intensified the issue. Additionally, greenhouse gas emissions generated throughout the plastic life cycle have heightened the urgency for sustainable recycling strategies [7]. Many government agencies, private organizations, and researchers have aimed to develop cost-effective materials to reduce environmental pollution. Therefore, they have investigated the feasibility and performance of using waste plastics in construction [8]. One of the plastic types whose applicability has been investigated is HDPE. Among polymers, HDPE represents a significant portion of global plastic production [5]. HDPE, commonly found in bottles, containers, and industrial packaging, is a prime example of non-biodegradable waste [9]. Using HDPE in cement-based composites helps reduce the accumulation of plastic waste on land and in the oceans. As emphasized in ref. [10], the “very low biodegradability” of plastic makes its recycling in construction a major environmental benefit. The physical and chemical structure of HDPE makes it suitable for use as a natural aggregate replacement in concrete [11]. There are studies highlighting that using HDPE in cementitious composites not only recycles waste but also improves the functional properties of concrete or mortar. For example, ref. [9] notes that using HDPE makes the concrete lighter and improves its insulation by lowering both density and thermal conductivity. This means that buildings made with HDPE-substituted concrete may use less energy overall.

Because HDPE is hydrophobic, it does not bond well to the surrounding materials when it is added to cement-based mixes [10,12]. Despite this drawback, the chemical inertness of HDPE provides resistance to degradation in alkaline cement environments [2]. When used as an aggregate, HDPE reduces compressive and tensile strength due to poor bonding. However, it also improves thermal insulation and reduces density [10,13]. This structure of HDPE is consistent with the work of ref. [14], which suggests that dry-cast blocks containing 3–6% polyethylene pellets achieve acceptable performance, but that using higher HDPE yields lower strength.

The use of HDPE as a fine aggregate has been the focus of several studies in the literature [15,16,17,18,19,20]. In a study ref. [20], HDPE was used to replace natural sand in cement mortar at replacement levels ranging from 0% to 100%. In this study, the authors examined density, compressive strength, modulus of elasticity, and stress–strain behavior. They showed that increasing the HDPE content reduced the density by up to 39% and the compressive strength by up to 83% at full replacement. However, mortars with a higher HDPE content exhibited higher ductility and deformation capacity compared to control samples.

Another study was conducted using HDPE powder from 5% to 30%, instead of fine aggregate in concrete, and 10% metakaolin instead of cement. Mechanical properties such as compressive, tensile, and flexural strength were tested. The best results were obtained from the mix with 15% HDPE and 10% metakaolin, which showed a 6% increase in compressive strength. When the HDPE content was increased beyond this level, the strength started to drop. However, water absorption decreased and durability improved as the amount of HDPE increased [17].

Earlier studies also explain where HDPE can and cannot be used. They indicate that HDPE cannot fully replace natural aggregates in structural concrete, which requires high load-carrying capacity. Still, HDPE offers clear benefits in applications where carrying heavy loads is not the main requirement. HDPE is considered a promising recycled material for sustainable construction. Its combination of durability, thermal insulation potential, and environmental benefits makes it a valuable candidate for further research in cement-based composites [21,22].

Previous investigations ref. [16,19,20] into the use of HDPE as a substitute in construction materials have introduced predictive models for mechanical performance. One such study developed equations demonstrating that the level of HDPE replacement directly affects both compressive strength and modulus of elasticity. The compressive strength relationship proposed by ref. [16] is expressed in Equation (1), while the modulus of elasticity relationship is given in Equation (2). In these equations, $f_{chdpe}^{\prime }$ denotes the compressive strength of specimens containing HDPE, $E_{chdpe}$ refers to the flexural strength of those specimens, and hdpe% indicates the percentage of HDPE incorporated as a replacement.

(1) $$f_{chdpe}^{\prime }=\left(f_{c}^{\prime }\right)\left(1-0.009026(hdpe\%)\right)$$

(2) $$E_{chdpe}=\left(E_{c}\right)\left(1-0.005522(hdpe\%)\right)$$

While some prior studies have demonstrated the mechanical behaviors of HDPE substitution, few have systematically integrated experimental and literature data into predictive frameworks. This study aims to fill that gap. In this research, up to 30% of the natural sand in concrete was replaced by volume with HDPE to promote the conservation of natural resources and enhance the recycling of HDPE. Alongside the experimental program, results from comparable studies in the literature were compiled, enabling a broader evaluation of how previous findings relate to the outcomes of the present work and helping to clarify the connections among the various experimental results.

## 2. Materials and Methods

**Figure 1:**
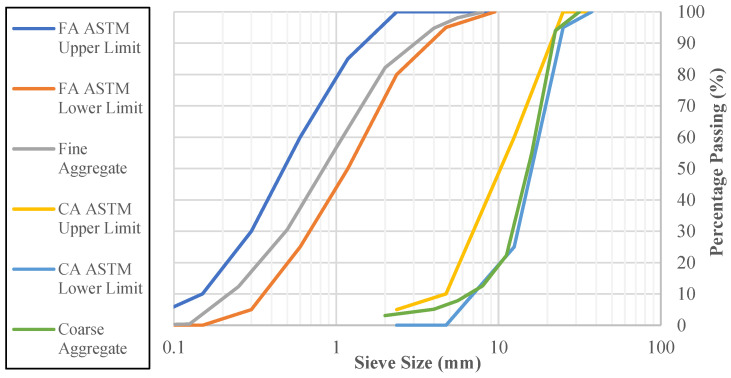
Particle size distribution curves of the aggregates.

Sieve analyses were performed separately on the coarse aggregate (crushed limestone) and fine aggregate (river sand) to determine their particle size distributions, in accordance with the procedures specified in the EN 933-1 [23] standard. The particle size distributions obtained from the sieving analyses are shown in Figure 1, which presents the grading curves for both coarse aggregates (CA) and fine aggregates (FA).

**Figure 2:**
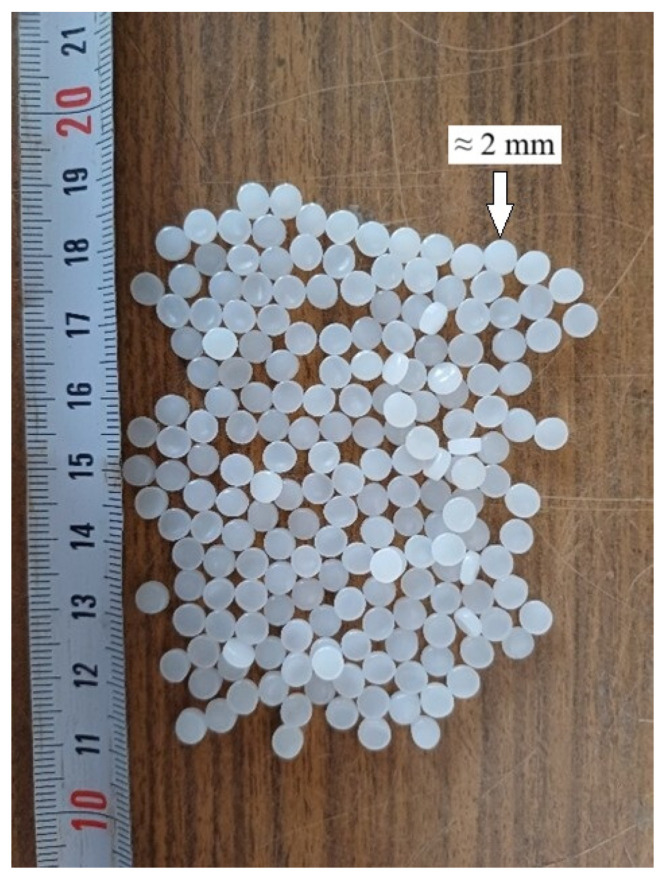
HDPE granules.

For the concrete mixtures, CEM I 42.5 R Portland cement was used. This cement meets the requirements of the EN 197-1 [24] standard. The high-density polyethylene (HDPE) granules used in the study were supplied by a plastic recycling facility in Turkey as part of their routine recycling process. A photograph of the HDPE granules is presented in Figure 2. The main physical and mechanical properties of these granules, together with those of the other aggregates used in the concrete, are given in Table 1.

**Figure 3:**
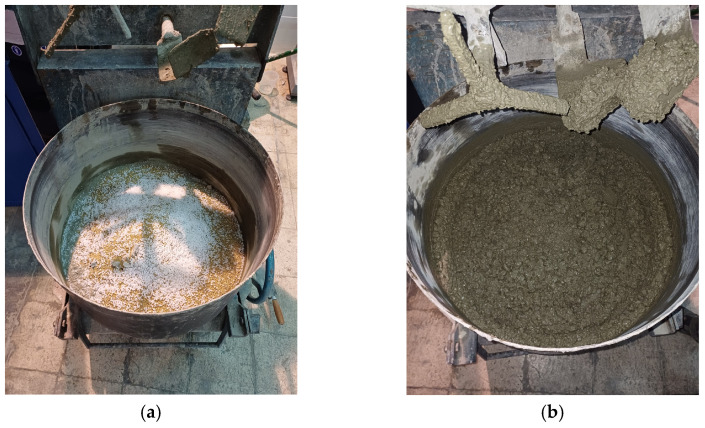
Concrete production: (**a**) before mixing, (**b**) after mixing.

The concrete specimens were produced in a mechanical mixer to achieve a uniform blend of the materials. In all mixtures, the amounts of water, cement, and coarse aggregate were kept the same to maintain consistency throughout the experimental study. The water-to-cement ratio was set at 0.5, which was considered appropriate in terms of both workability and strength. In the mixtures containing high-density polyethylene (HDPE) granules, the fine aggregate (sand) was partly replaced at three levels, 10%, 20%, and 30% by volume. The HDPE granules had a uniform particle size of about 2 mm, which slightly affects fine aggregate gradation, but at these low substitution levels, the impact remains acceptable for experimental evaluation. Figure 3 presents images related to the concrete production process.

To keep the specimens easily distinguishable during testing, a simple naming scheme was used. Specimens tested for compressive strength were labeled “CS”, those for splitting tensile strength were marked “STS”, and the flexural strength samples were labeled “FS”. Mixes that contained HDPE included “HDPE” in their name, while the control samples without HDPE were labeled “R”. The percentage of HDPE replacement was also added to each specimen’s label. A summary of the mixture details is given in Table 2.

The experimental program was structured to generate a consistent baseline dataset for validating predictive models and to enable comparison with data reported in the literature. Concrete mixtures were prepared using a mixing machine. A slump test was performed to measure the workability of the fresh concrete. This test followed standard procedures using a conical mold. The mold was filled halfway and compacted with 25 strokes of a tamping rod. Then it was filled to the top, and another 25 strokes were applied. The surface was leveled, and the mold was lifted straight up so the concrete could slump under its own weight. The difference between the original height and the final height was recorded as the slump value.

Cylindrical specimens (100 mm in diameter and 200 mm in height) were made for compressive and splitting tensile strength tests. Prismatic beams (100 × 100 × 400 mm) were produced for flexural strength testing. The target compressive strength of the reference concrete was 25 MPa. All specimens were cured in water for 28 days. After curing, the samples were dried, weighed, and their densities were calculated.

**Figure 4:**
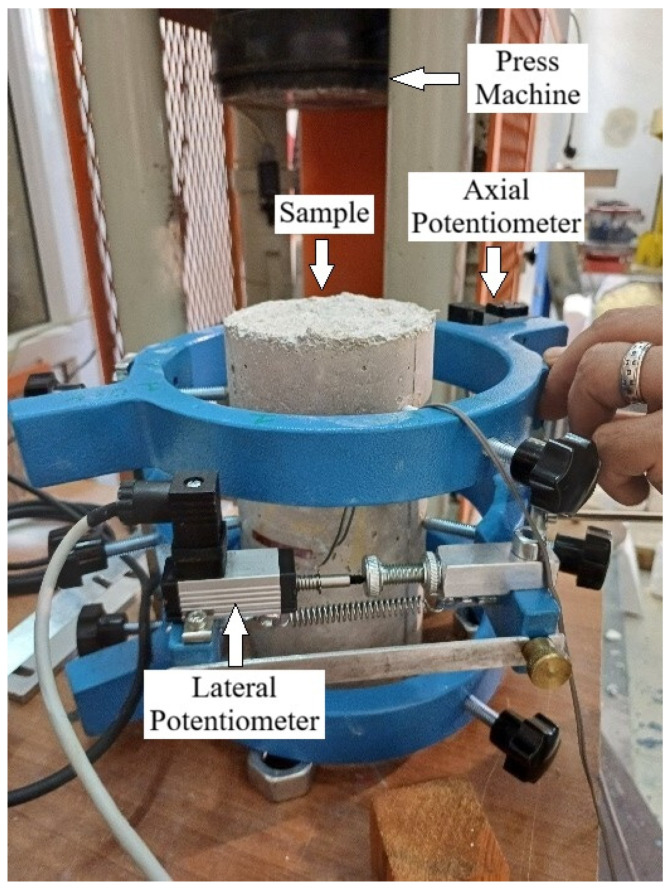
Experimental setup for the compressive strength test.

For the compressive strength test, the cylindrical samples were fitted with potentiometers and strain gauges to measure axial and lateral deformation. The specimens were placed in a hydraulic testing machine, and axial load was applied at a constant rate of 0.5 MPa. Stress was calculated by dividing the applied load by the cross-sectional area. Axial strain was found by dividing the average axial displacement by the height of the specimen. Lateral strain was found by dividing the lateral displacement by the specimen diameter. The test setup is shown in Figure 4.

For the splitting tensile strength test, a loading cage was used to ensure the cylinders were loaded properly. Each sample was placed horizontally in the testing machine and loaded at a constant rate of 0.5 MPa. The maximum load the sample carried before it failed was recorded as its splitting tensile strength.

Flexural strength was measured using prismatic beams tested with the three-point bending method. The load was applied at a rate of 5 mm/min, and the distance between the supports was set to 300 mm.

## 3. Concrete Tests

Experiments conducted on concrete samples yielded various properties, including slump and density, compressive strength, splitting tensile strength, flexural strength, and modulus of elasticity. Details of the experimental findings are presented in the following subsections.

### 3.1. Compressive Strength Test

**Figure 5:**
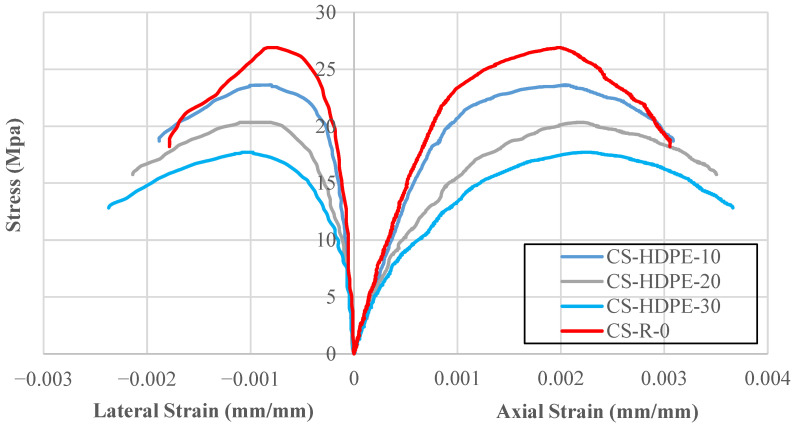
Stress–strain behavior of concrete specimens.

Stress–strain curves obtained from compressive strength tests are presented in Figure 5, and the maximum compressive strength ($f_{co}^{\prime }$), axial strain ($\varepsilon_{co}$), and lateral strain at peak stress ($\varepsilon_{lo}$) values are compiled in Table 3. The compressive strength of the reference concrete without HDPE was measured as 26.91 MPa and was used as the basis for evaluating other mixtures.

As the use of HDPE as a fine aggregate replacement ratio increased, compressive strength decreased by 12.20% to 23.63 MPa. As the replacement ratio increased to 20%, the decrease in strength became even more pronounced, decreasing by 24.43% to 20.33 MPa. The largest decrease was recorded at a 30% substitution ratio, where strength decreased by 34.19% to 17.71 MPa. These results clearly show that the use of polymer-based materials has a negative effect on the load-carrying capacity of the concrete matrix.

Although the compressive strength decreased, the axial and lateral strain values increased noticeably as the HDPE content increased. HDPE-substituted mixtures could deform more before failing. Axial strain increased by 4.42%, 9.34%, and 16.34% at 10%, 20%, and 30% HDPE replacement levels. Lateral strain increased by 7.28%, 34.12%, and 43.81% at the same levels. HDPE reduces the stiffness of the concrete; it also makes the material more ductile.

The decrease in compressive strength is caused by the weaker bond between HDPE and the cement, compared to the natural aggregates. But due to the increased strain values, HDPE allows the concrete to deform more before it breaks. This can be useful in situations where high strength is not the main priority, but deformation capacity is important. While the incorporation of HDPE reduces ultimate compressive strength, the resulting enhancement in strain capacity makes it an ideal candidate for non-structural and semi-structural applications. These include lightweight partition walls, pavement blocks, sidewalks, and precast garden furniture, where high load-bearing capacity is not a primary requirement.

### 3.2. Splitting Tensile Strength Test

The splitting tensile strength results are shown in Table 4. The reference mix without HDPE had a strength of 2.46 MPa. As the HDPE content increased, the splitting tensile strength gradually decreased. At the 10% replacement level, the strength dropped by about 7.6%, reaching 2.28 MPa. The decrease became more pronounced as the substitution rate increased to 20%, with strength decreasing by 15.77% to 2.07 MPa. The largest decrease was observed at a 30% substitution level, where splitting tensile strength decreased by 22.55%, reaching 1.91 MPa.

### 3.3. Flexural Strength Test

**Figure 6:**
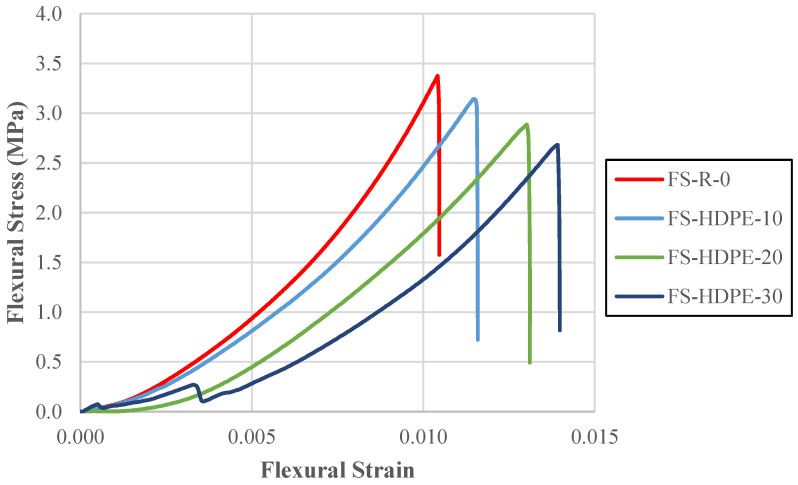
Flexural stress–flexural strain behavior of concrete specimens.

The flexural behavior of the concrete samples was evaluated through flexural tests performed using the three-point loading principle; the resulting stress–strain curves are presented in Figure 6. The numerical results for flexural strength and maximum strain values are summarized in Table 5. The flexural strength of the reference concrete mix without HDPE additives was determined to be 3.37 MPa and was used as the basis for evaluating the performance of the modified mixes.

As the amount of HDPE used to replace fine aggregate increased, the flexural strength decreased. At 10% replacement, it dropped by 6.86% to 3.14 MPa. At 20%, the strength fell further by 14.51% to 2.88 MPa. At 30%, it decreased by 20.56% to 2.68 MPa. HDPE reduces the concrete’s ability to resist flexural stresses. The reduction in flexural strength, as observed with compressive strength, arises from the weaker bond between HDPE and cement relative to natural aggregates.

The maximum strain values increased as more HDPE was replaced. At 10% replacement, strain increased by 10.74%. At 20% replacement, the increase reached 25.24%, and at 30% replacement, it rose by 33.60%. This indicates that although the flexural strength decreases, concrete containing HDPE can deform more before failure, giving it greater flexibility under flexural loads.

### 3.4. Modulus of Elasticity Test

The modulus of elasticity values from the compressive strength tests are shown in Table 6. These values were calculated using the stress–strain curves and were used to evaluate the stiffness of the mixtures. The reference concrete without HDPE had a modulus of elasticity of 30,574.17 MPa.

As the amount of HDPE replacing fine aggregate increased, the modulus of elasticity showed a clear and consistent decrease. At 10% replacement, it dropped by 15.00% to 25,987.98 MPa. This reduction became more significant at 20% replacement, where stiffness fell by 30.45% to 21,264.47 MPa. The largest decrease occurred at 30% HDPE, with the modulus dropping by 33.79% to 20,241.88 MPa.

### 3.5. Slump Test

**Figure 7:**
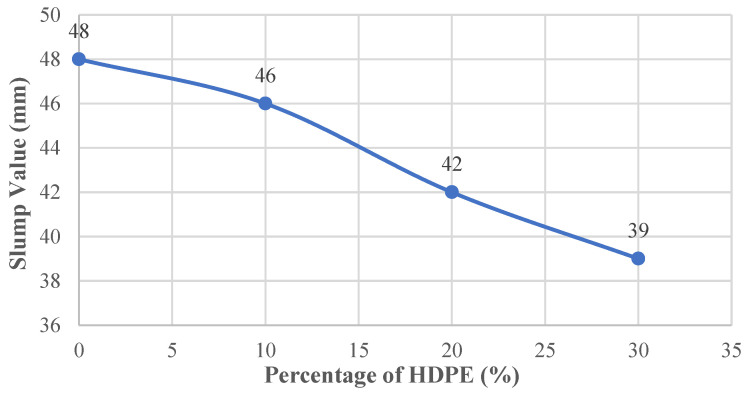
Slump value–HDPE content curve.

The slump test results for the concrete mixtures are shown in Figure 7. The reference mix without HDPE had a slump value of 48 mm, which represents the normal workability of conventional concrete and was used as the baseline for comparison.

As HDPE replaced a larger portion of the fine aggregate, the slump values consistently decreased. When 10% of the sand was replaced with HDPE, the slump dropped by 4.17% to 46 mm. At 20% replacement, the slump decreased more noticeably, falling by 12.50% to 42 mm. The largest reduction occurred at the 30% level, where the slump value decreased by 18.75% and reached 39 mm.

### 3.6. Density Test

**Figure 8:**
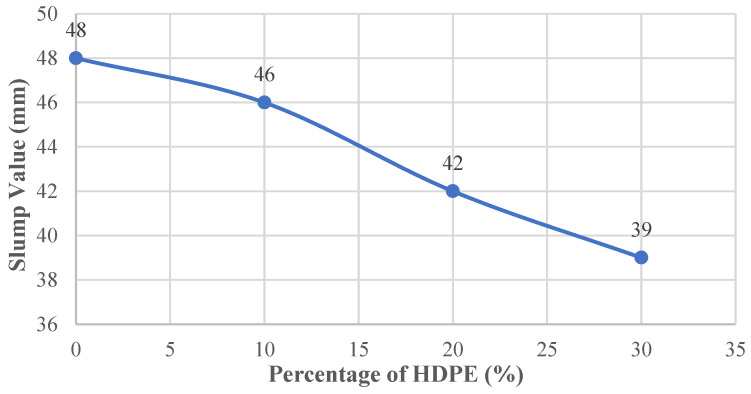
Density value–HDPE content curve.

The density results for the concrete mixes are shown in Figure 8. The reference mix without HDPE had a density of 2307.88 kg/m^3^, which represents the typical unit weight of conventional concrete and was used as the baseline for comparison.

As more HDPE granules were used to replace fine aggregate, the density of the concrete gradually decreased. With 10% sand replacement, the density dropped by 3.74% to 2221.54 kg/m^3^. At 20% replacement, the decrease became more noticeable, falling by 5.44% to 2182.35 kg/m^3^. The largest reduction occurred at the 30% level, where the density decreased by 8.44% to 2113.18 kg/m^3^.

## 4. Compilation of Experimental Data and Formulation of Models

In this section, a comprehensive database was created to help develop and evaluate predictive models for concrete and mortar containing HDPE. The database was built by systematically reviewing previous studies on concrete mixes where high-density polyethylene (HDPE) partially replaces fine aggregate. The experimental results from this study were also added to expand the dataset and make it more useful. The combined experimental results for HDPE-reinforced concrete and mortar are summarized in Table 7.

Using this database, several models were proposed to predict key mechanical and physical properties of HDPE-substituted concrete and mortar, including compressive strength, tensile strength, flexural strength, and modulus of elasticity. The accuracy of these new models was tested by comparing them with models from previous studies.

The agreement between model predictions and experimental results was calculated using statistical measures summarized in Table 8. Table 8 includes the coefficient of determination (R^2^), the mean absolute bias error (MABE), and the mean absolute percentage error (MAPE). R^2^ shows how well the predicted values match the experimental data, indicating how much of the variation in the data the model can explain. MABE measures the average difference between predicted and experimental values, while MAPE expresses this difference as a percentage, making it easier to understand accuracy across different scales [26].

In Table 8, ${exp}_{i}$ refers to individual experimental results, and ${mod}_{i}$ refers to the corresponding model predictions. The terms ${\bar{exp}}_{i}$ and ${\bar{mod}}_{i}$ represent the averages of the experimental results and model predictions.

### 4.1. Compressive Strength Model Formulation

Compressive strength results from previous studies are summarized in Table 8. To see how HDPE affects compressive strength, the strength of HDPE-substituted concrete was divided by the strength of the reference concrete. The compressive strength ratio ($\frac{f_{chdpe}^{\prime }}{f_{c}^{\prime }}$) was found where ($f_{chdpe}^{\prime }$) represents the compressive strength of the HDPE-substituted concrete, and ($f_{c}^{\prime }$) represents the compressive strength of the reference concrete.

**Figure 9:**
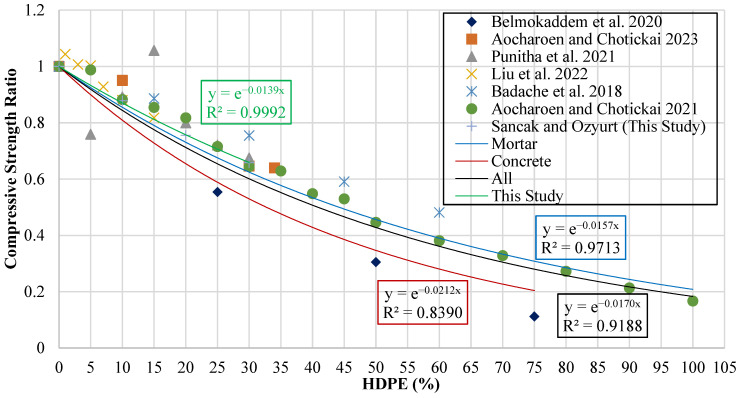
Curve of compressive strength ratio versus HDPE content [15,16,17,18,19,20].

The ratio is shown in a graph, with the vertical axis representing the compressive strength ratio and the horizontal axis showing the HDPE replacement percentage (hdpe%). Values above 1 mean that HDPE has a positive effect on compressive strength, while values below 1 indicate a negative effect. The graph is presented in Figure 9.

To analyze the data, trend lines were applied to the obtained results. These trend lines were created separately for concrete mixes, mortar mixes, experimental data from the current study, and the combined dataset encompassing all results. As part of this process, predictive model proposals were developed for each category. Exponential trend lines were observed to provide a better fit than linear or polynomial alternatives and to more accurately reflect the correlation between the HDPE replacement percentage and the compressive strength ratio. The equations from exponential trend lines are shown in Table 9.

The models were evaluated using statistical indicators calculated from the combined experimental data. The results of this evaluation, along with the model equations, are summarized in Table 10. This table clearly shows how closely model predictions agree with the experimental data. In the table, the parameter $\gamma_{chdpe}$ represents the density of the HDPE-substituted concrete.

The predictive model developed by ref. [19] used the density parameter $\gamma_{chdpe}$ as a key factor for estimating compressive strength. Including density made the model physically meaningful, but its use was limited because some studies in the compiled database did not report $\gamma_{chdpe}$ values. This meant fewer data points were available to test the model.

**Figure 10:**
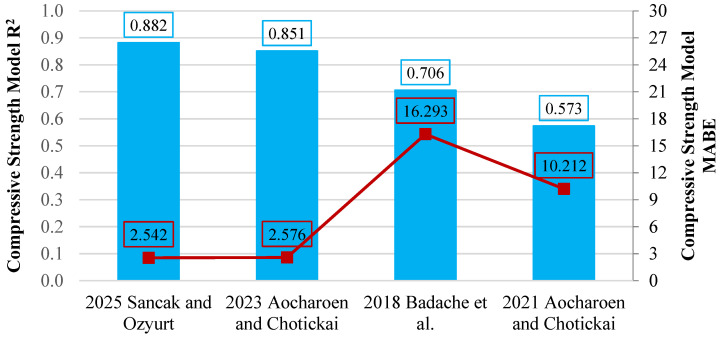
Evaluation of compressive strength models using R^2^ and MABE [16,19,20].

The proposed model provides the most accurate predictions, with an R^2^ of 0.882. This high value indicates that the Sancak and Ozyurt model effectively captures the influence of HDPE content on compressive strength, performing better than other models. Ref. [16] follows with an R^2^ of 0.851, demonstrating good predictive reliability. In contrast, the earlier model [20] only achieved an R^2^ of 0.574, indicating low accuracy and limited applicability for HDPE-substituted concrete. A graphical comparison of the statistical performance of the different models is presented in Figure 10, which shows R^2^ and MABE values.

### 4.2. Tensile Strength Model Formulation

**Figure 11:**
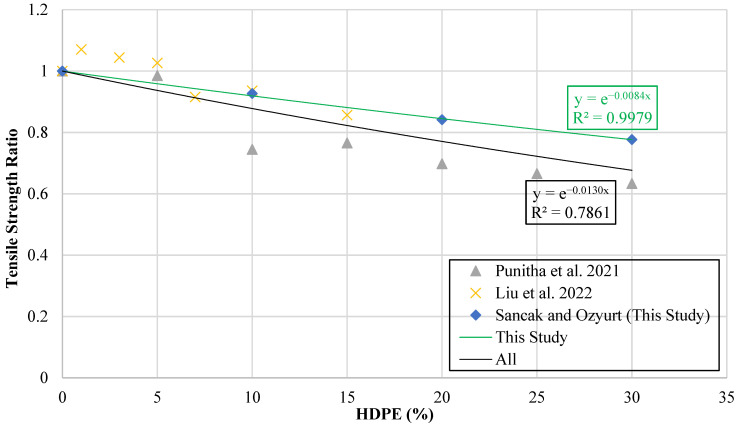
Curve of tensile strength ratio versus HDPE content [17,18].

In the development of the tensile strength models, a methodology analogous to that employed for the compressive strength analysis was adopted. Specifically, the experimental data were processed to generate a graphical representation in which the vertical axis corresponds to the tensile strength ratio ($\frac{f_{thdpe}^{\prime }}{f_{t}^{\prime }}$), while the horizontal axis represents the percentage of HDPE substitution (hdpe%). This ratio was selected to normalize the tensile strength of HDPE-modified mixtures against the reference concrete, thereby enabling a direct comparison of performance across different substitution levels. The resulting graph is presented in Figure 11.

Trendlines were fitted to the plotted data in order to identify mathematical relationships between HDPE substitution percentage and tensile strength ratio. Separate trendlines were generated for two datasets: (i) substitution in the experimental results obtained in the present study and (ii) the combined dataset incorporating all available data (substitution in concrete mixtures). From these trend lines, new model proposals were formulated to predict the tensile strength behavior of HDPE-substituted systems.

The equations and proposed models resulting from this process are summarized in Table 11. These models provide a quantitative framework for estimating the reduction in tensile strength of concrete depending on the HDPE content. Table 11 also explains the symbols used during modeling: $f_{thdpe}^{\prime }$ is the tensile strength of HDPE-substituted concrete, $f_{t}^{\prime }$ is the tensile strength of the reference (non-HDPE-substituted) concrete, and hdpe% represents the percentage of HDPE particles used to replace fine aggregate.

The existing literature review revealed that no previously published model directly addresses the relationship between the HDPE replacement ratio and the tensile strength of concrete. To test the reliability of the proposed models, their applicability was evaluated using statistical performance indicators derived from a consolidated experimental database. The numerical results of these analyses are presented in Table 11.

Numerous studies in the literature have examined the relationship between the compressive and tensile strength of concrete, and most have proposed empirical models to describe this relationship. The current study incorporated these previous approaches into its analysis and attempted to develop comparable models using the compiled experimental database.

**Figure 12:**
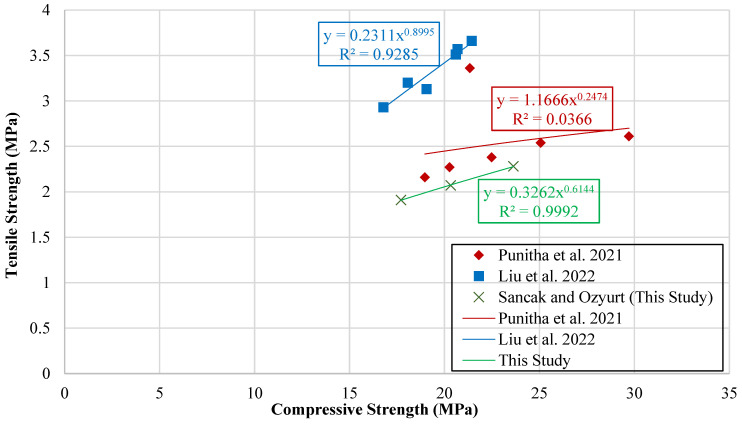
Curve of tensile strength versus compressive strength [17,18].

A graph was created to compare compressive strength with tensile strength. When preparing the dataset, care was taken to account for differences in sample shapes between studies. Compressive strength was usually measured on cubes, while tensile strength was measured on cylinders. To make the results comparable, cube strength values were converted to equivalent cylinder strength, removing any inconsistencies caused by differences in specimen shape and size. This graph is presented in Figure 12.

Trendlines were then fitted to the plotted data to capture the underlying relationship between the two strength parameters. These fitted trendlines served as the basis for generating new model proposals, which were designed to predict tensile strength as a function of compressive strength. The mathematical equations derived from these trendlines, along with the corresponding model formulations, are presented in Table 12.

Trendlines were also generated for the experimental data reported in other studies, in addition to those obtained in the present investigation. However, a unified model proposal that encompassed all datasets was not introduced, as the combined data were considered inconsistent due to the limited number of available results.

### 4.3. Flexural Strength Model Formulation

**Figure 13:**
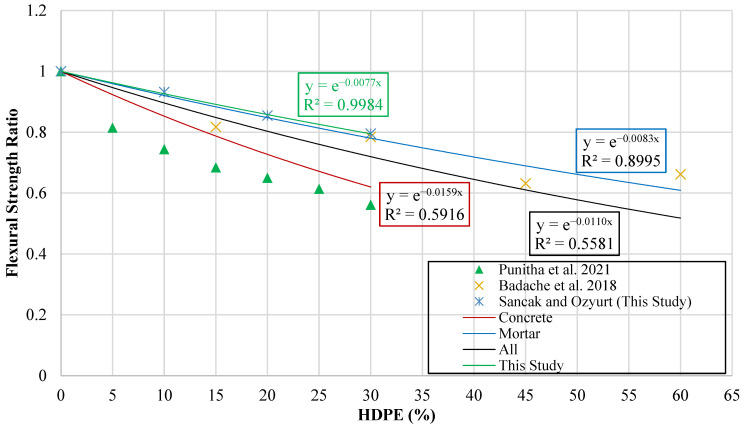
Curve of flexural strength ratio versus HDPE content [17,19].

For the evaluation of flexural strength, a graph was constructed in which the vertical axis represents the flexural strength ratio ($\frac{f_{rhdpe}^{\prime }}{f_{r}^{\prime }}$), while the horizontal axis corresponds to the HDPE substitution percentage (hdpe%). This ratio was employed to normalize the flexural strength of HDPE-modified mixtures against the reference concrete, thereby enabling a direct comparison across different substitution levels. The resulting plot is presented in Figure 13.

Using the data, trendlines were fitted to the graph in order to identify mathematical relationships between substitution percentage and flexural strength ratio. Separate model suggestions were generated for substitution in concrete, substitution in mortar, the experimental results obtained in the present study, and the combined dataset incorporating all available results. These fitted trendlines provided the basis for deriving predictive equations, which were subsequently proposed as models for estimating the flexural strength behavior of HDPE-substituted concrete.

The equations obtained through this process, together with the developed models, are summarized in Table 13. In this table, $f_{rhdpe}^{\prime }$ denotes the flexural strength of HDPE-substituted concrete, $f_{r}^{\prime }$ represents the flexural strength of the reference (unsubstituted) concrete, and hdpe% indicates the percentage of HDPE substitution.

**Figure 14:**
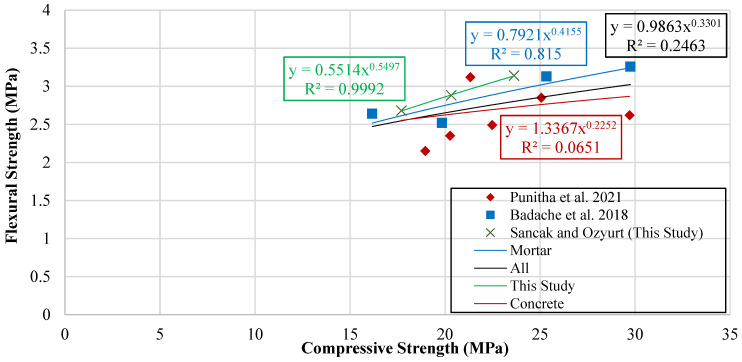
Curve of flexural strength versus compressive strength [17,19].

Several studies have investigated the relationship between compressive strength and flexural strength in concrete and proposed empirical models. Building on these contributions, the present study constructed a graph in Figure 14, plotting compressive strength against flexural strength. Trendlines fitted to the data were then used to generate new predictive models, which estimate flexural strength as a function of compressive strength. The resulting equations and models are presented in Table 14.

### 4.4. Modulus of Elasticity Model Formulation

**Figure 15:**
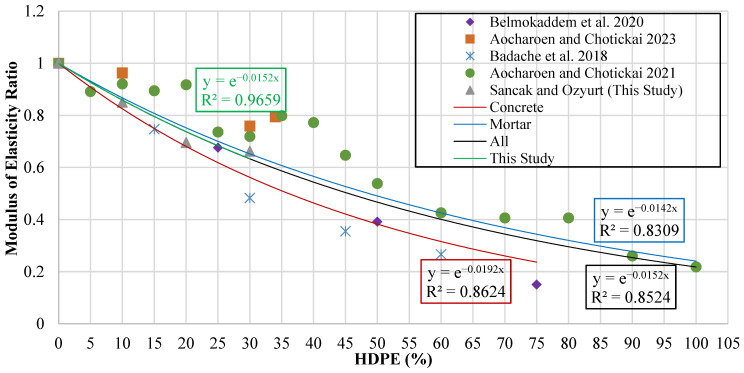
Curve of modulus of elasticity ratio versus HDPE content [15,16,19,20].

For the evaluation of the elasticity modulus, a graph was constructed in which the vertical axis represents the elasticity modulus ratio ($\frac{E_{chdpe}}{E_{c}}$), while the horizontal axis corresponds to the HDPE substitution percentage (hdpe%). This ratio was employed to normalize the modulus of HDPE-modified mixtures against the reference concrete, thereby enabling direct comparison across different substitution levels. The resulting plot is presented in Figure 15.

Trendlines were fitted to the data points in order to identify the mathematical relationship between HDPE substitution and modulus of elasticity. Based on these fitted curves, model proposals were generated for four categories: substitution in concrete, substitution in mortar, experimental results obtained in the present study, and the combined dataset, including all available data. Unlike earlier studies relying on linear formulations, the proposed models employ exponential equations to better represent the nonlinear strength loss behavior of HDPE-modified concrete.

The equations obtained from these trend lines and the corresponding model formulations are summarized in Table 15. The table shows $E_{chdpe}$ the modulus of elasticity of HDPE-substituted concrete, $E_{c}$ the modulus of elasticity of the reference (without HDPE substitution) concrete, and the proportion of HDPE substituted with hdpe%.

The models developed in this study were evaluated for applicability against models available in the literature to predict the relationship between modulus of elasticity and compressive strength in HDPE-reinforced concrete. Validation was conducted using statistical performance indicators obtained from an experimental database, and the accuracy of the model predictions was objectively compared. The results of the relevant statistical analyses and model formulations are presented in Table 16.

**Figure 16:**
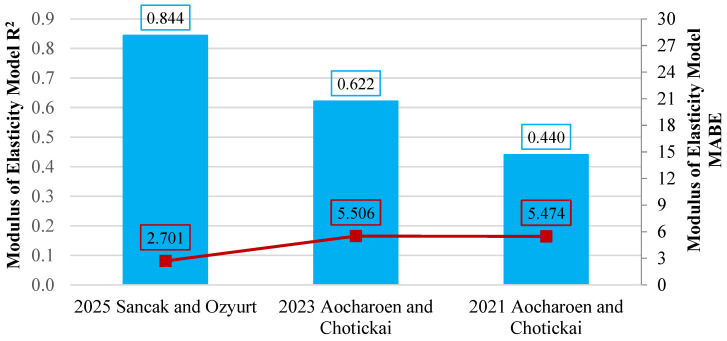
Evaluation of models linking modulus of elasticity to HDPE content using R^2^ and MABE [16,20].

Statistical analyses showed that the model proposed in this study provided the highest predictive accuracy, with an R^2^ of 0.844. In contrast, the reference [20] model performed quite poorly, achieving an R^2^ value of only 0.440, indicating limited reliability and low predictive capacity. A comparative presentation of the obtained results is given in Figure 16, which includes the R^2^ and mean absolute error of bias (MABE) values of the evaluated models.

**Figure 17:**
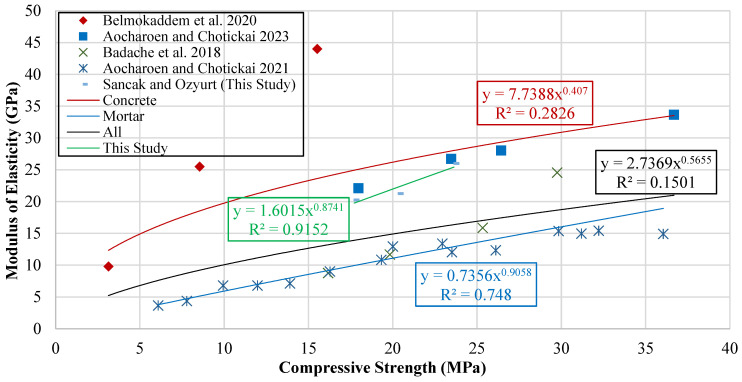
Curve of modulus of elasticity versus compressive strength [15,16,19,20].

Numerous studies in the existing literature investigate the relationship between the compressive strength and modulus of elasticity of concrete, and various empirical models have been developed to explain this relationship. Based on these previous studies, this research provides a comparative assessment, and a graph showing the relationship between compressive strength values and the corresponding modulus of elasticity is presented in Figure 17.

Trend lines were added to the results to show the basic relationship between the modulus of elasticity and compressive strength. The equations from these trend lines are given in Table 17.

The developed models were compared with similar models from the literature to evaluate their accuracy. This comparison was based on statistical performance indicators, and the results are summarized in Table 18.

**Figure 18:**
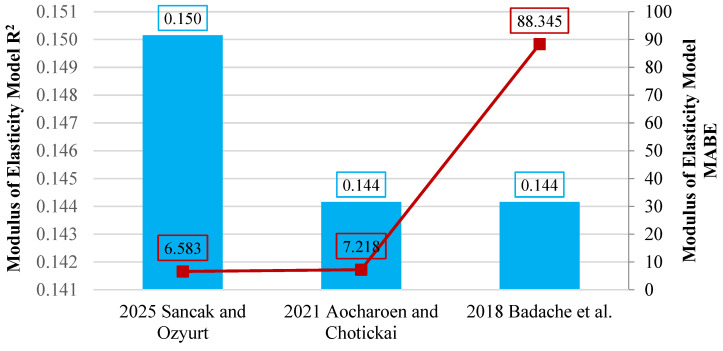
Evaluation of models linking modulus of elasticity to compressive strength using R^2^ and MABE [19,20].

The performance assessment showed that the model developed in this study outperformed the remaining models, yielding an R^2^ value of 0.150. Even though this value indicates a weak correlation, it is still greater than those obtained from the other alternatives. Additionally, the model achieved the most favorable MABE result, with a value of 6.583, whereas the ref. [19] model demonstrated significantly lower accuracy, reflected by its much higher MABE of 88.345. Figure 18 presents the comparative plots of the R^2^ and MABE values for all evaluated models.

Exponential equations were found to provide the most consistent fit when modeling the effects of HDPE substitution percentage on compressive strength, splitting tensile strength, flexural strength, and modulus of elasticity. In contrast, power equations yielded better consistency when estimating splitting tensile strength, flexural strength, and modulus of elasticity from compressive strength.

Some of the models presented here, especially those developed exclusively from the experimental data of this study, are constrained by the limited number of data points. Expanding the dataset in future investigations will be essential to minimize misfitting and improve the general applicability of the results. The models proposed can then be updated and recalibrated to achieve greater accuracy.

## 5. Conclusions

In this study, the influence of incorporating high-density polyethylene (HDPE) as a partial replacement for fine aggregate in concrete mixtures was systematically investigated with respect to a range of mechanical and physical properties. To support this study, an experimental database was created by combining the results from this research with data from previous studies. This database served as a foundation for new predictive models and for testing the accuracy of existing models from the literature.

Using HDPE granules as a substitute reduces the workability of fresh concrete. The decrease in slump values indicates that mix adjustments, such as adding plasticizers or modifying the water–cement ratio, may be needed at higher HDPE content. Nevertheless, a substitution level of 10% has been shown to maintain workability within acceptable limits.

Increasing the amount of HDPE in the mixes reduced the density of the concrete. HDPE can aid in the production of lightweight concrete and may be useful in applications where reducing dead load is important.

HDPE caused noticeable decreases in the modulus of elasticity, compressive strength, tensile splitting strength, and flexural strength. However, the ultimate strain increased, making the concrete more deformable before cracking. HDPE-replaced concrete may be suitable for situations where ductility is more important than ultimate strength.

The predictive models developed in this study agreed well with the experimental results and outperformed other models examined. However, further studies are needed to better understand long-term performance and structural behavior at higher HDPE replacement levels.

The accuracy of the models in this study depends on the quantity and quality of available data. While significant effort has been made to compile information from various studies, differences in testing procedures and reporting may affect model reliability. Models may need to be updated as new data is added.

HDPE substitution offers both advantages and disadvantages. It can make concrete lighter and more ductile, but it also reduces strength and stiffness. The models developed here provide a useful tool for predicting these effects. However, it is emphasized that more research is needed to understand the behavior of HDPE-substituted concrete.

## Figures and Tables

**Table 1:** Aggregate properties and HDPE granule properties.

	HDPE	Coarse	Fine
Specific Gravity	0.95	2.7	2.6
Unit Weight (kg/m^3^)	946	1950	1800
Size (mm)	2	2–22.4	0–8

**Table 2:** Mix designs and specimen details.

Strength Test	Water (kg/m^3^)	Cement (kg/m^3^)	Crushed Limestone (kg/m^3^)	River Sand (kg/m^3^)	HDPE (kg/m^3^)	HDPE Content Vol (%)	Sample Name
Compressive	195	390	1060	730	-	-	CS-R-0
				657	38.37	10	CS-HDPE-10
				584	76.73	20	CS-HDPE-20
				511	115.10	30	CS-HDPE-30
Splitting tensile	195	390	1060	730	-	-	STS-R-0
				657	38.37	10	STS-HDPE-10
				584	76.73	20	STS-HDPE-20
				511	115.10	30	STS-HDPE-30
Flexural	195	390	1060	730	-	-	FS-R-0
				657	38.37	10	FS-HDPE-10
				584	76.73	20	FS-HDPE-20
				511	115.10	30	FS-HDPE-30

**Table 3:** Compressive strength performance of the concrete specimens.

Sample Name	Maximum Compressive Stress ($f_{co}^{\prime }$) (MPa)	Axial Strain ($\varepsilon_{co}$) at Maximum Stress	Lateral Strain ($\varepsilon_{lo}$) at Maximum Stress
CS-R-0	26.91	0.001967	0.000747
CS-HDPE-10	23.63	0.002054	0.000801
CS-HDPE-20	20.33	0.002151	0.001002
CS-HDPE-30	17.71	0.002289	0.001074

**Table 4:** Split tensile strength performance of the concrete specimens.

Sample Name	Split Tensile Strength (MPa)
CS-R-0	2.46
CS-HDPE-10	2.28
CS-HDPE-20	2.07
CS-HDPE-30	1.91

**Table 5:** Flexural strength performance of the concrete specimens.

Sample Name	Maximum Flexural Stress (MPa)	Maximum Flexural Strain
CS-R-0	3.37	0.010467
CS-HDPE-10	3.14	0.011591
CS-HDPE-20	2.88	0.013109
CS-HDPE-30	2.68	0.013983

**Table 6:** Modulus of elasticity of the specimens.

Sample Name	Modulus of Elasticity (MPa)
CS-R-0	30,574.17
CS-HDPE-10	25,987.98
CS-HDPE-20	21,264.47
CS-HDPE-30	20,241.88

**Table 7:** Experimental database of HDPE-substituted samples.

Type of Material	Type of Substitution	Year	Reference	28-Day Compressive Strength Test (mm)	28-Day Tensile Strength Test (mm)	28-Day Flexural Strength Test (mm)	HDPE Specific Gravity	HDPE Bulk Density (kg/m3)	HDPE Size (mm)	HDPE (%)	Concrete Density (kg/m3)	W/C Ratio	28-Day Compressive Strength (MPa)	28-Day Cylindrical CS (MPa)	28-Day Tensile Strength (MPa)	28-Day Flexural Strength (MPa)	Modulus of Elasticity (GPa)	Slump (mm)
Concrete	Vol	2020	Belmokaddem et al. [15]	100 cubes						0	2438.14	0.48	35.02	28.02			65.1	
				100 cubes			0.96		0–8	25	2124.01	0.48	19.4	15.52			44	
				100 cubes			0.96		0–8	50	1772.66	0.48	10.68	8.54			25.5	
				100 cubes			0.96		0–8	75	1400.61	0.48	3.92	3.14			9.8	
Concrete	Vol	2023	Aocharoen and Chotickai [16]	100 × 200 cylinders						0		0.62	27.81	27.81			29.1	100
				100 × 200 cylinders			0.964		2–5	10		0.62	26.43	26.43			28.02	64
				100 × 200 cylinders			0.964		2–5	30		0.62	17.97	17.97			22.09	44
				100 × 200 cylinders						0		0.47	36.68	36.68			33.65	88
				100 × 200 cylinders			0.964		2–5	34		0.47	23.47	23.47			26.73	47
Concrete	Vol	2025	Sancak and Ozyurt (This Study)	100 × 200 cylinders	100 × 200 cylinders	100 × 100 × 400				0	2307.88	0.5	26.91	26.91	2.46	3.37	30.57	48
				100 × 200 cylinders	100 × 200 cylinders	100 × 100 × 400	0.95	946	2	10	2221.54	0.5	23.63	23.63	2.28	3.14	25.99	46
				100 × 200 cylinders	100 × 200 cylinders	100 × 100 × 400	0.95	946	2	20	2182.35	0.5	20.33	20.33	2.07	2.88	21.26	42
				100 × 200 cylinders	100 × 200 cylinders	100 × 100 × 400	0.95	946	2	30	2113.18	0.5	17.71	17.71	1.91	2.68	20.24	39
Concrete	Wei	2021	Punitha et al. [17]	150 cubes	150 × 300 cylinders	100 × 100 × 500				0		0.45	35.14	28.11	3.41	3.83		32
				150 cubes	150 × 300 cylinders	100 × 100 × 500	0.93			5		0.36	26.67	21.34	3.36	3.12		27
				150 cubes	150 × 300 cylinders	100 × 100 × 500	0.93			10		0.36	31.33	25.06	2.54	2.85		23
				150 cubes	150 × 300 cylinders	100 × 100 × 500	0.93			15		0.36	37.14	29.71	2.61	2.62		21
				150 cubes	150 × 300 cylinders	100 × 100 × 500	0.93			20		0.36	28.1	22.48	2.38	2.49		18
				150 cubes	150 × 300 cylinders	100 × 100 × 500	0.93			25		0.36	25.33	20.26	2.27	2.35		14
				150 cubes	150 × 300 cylinders	100 × 100 × 500	0.93			30		0.36	23.71	18.97	2.16	2.15		12
Concrete	Wei	2022	Liu et al. [18]	60 × 120 cylinders	60 × 120 cylinders					0		0.55	20.54	20.54	3.42			
				60 × 120 cylinders	60 × 120 cylinders				2–4	1		0.55	21.43	21.43	3.66			
				60 × 120 cylinders	60 × 120 cylinders				2–4	3		0.55	20.68	20.68	3.57			
				60 × 120 cylinders	60 × 120 cylinders				2–4	5		0.55	20.6	20.6	3.51			
				60 × 120 cylinders	60 × 120 cylinders				2–4	7		0.55	19.06	19.06	3.13			
				60 × 120 cylinders	60 × 120 cylinders				2–4	10		0.55	18.07	18.07	3.2			
				60 × 120 cylinders	60 × 120 cylinders				2–4	15		0.55	16.79	16.79	2.93			
Mortar	Vol	2018	Badache et al. [19]	EN 196-1 [25]		EN 196-1 [25]				0	2106.9	0.5	41.98	33.58		3.99	32.86	
				EN 196-1		EN 196-1	0.922	362	<3.15	15	1994.71	0.5	37.19	29.75		3.26	24.55	
				EN 196-1		EN 196-1	0.922	362	<3.15	30	1849.43	0.5	31.67	25.34		3.13	15.86	
				EN 196-1		EN 196-1	0.922	362	<3.15	45	1696.78	0.5	24.79	19.83		2.52	11.66	
				EN 196-1		EN 196-1	0.922	362	<3.15	60	1577.24	0.5	20.21	16.17		2.64	8.76	
Mortar	Vol	2021	Aocharoen and Chotickai [20]	50 cubes						0	2398.37	0.485	45.63	36.50			16.74	
				50 cubes			0.964		2–4.75	5	2373.98	0.485	45.06	36.05			14.92	
				50 cubes			0.964		2–4.75	10	2300.81	0.485	40.27	32.22			15.41	
				50 cubes			0.964		2–4.75	15	2284.55	0.485	38.99	31.19			14.97	
				50 cubes			0.964		2–4.75	20	2260.16	0.485	37.3	29.84			15.36	
				50 cubes			0.964		2–4.75	25	2203.25	0.485	32.64	26.11			12.32	
				50 cubes			0.964		2–4.75	30	2162.6	0.485	29.39	23.51			12.04	
				50 cubes			0.964		2–4.75	35	2097.56	0.485	28.68	22.94			13.37	
				50 cubes			0.964		2–4.75	40	2056.91	0.485	25.01	20.01			12.93	
				50 cubes			0.964		2–4.75	45	2032.52	0.485	24.16	19.33			10.83	
				50 cubes			0.964		2–4.75	50	1959.35	0.485	20.35	16.28			9.01	
				50 cubes			0.964		2–4.75	60	1878.05	0.485	17.38	13.90			7.13	
				50 cubes			0.964		2–4.75	70	1772.36	0.485	14.97	11.98			6.8	
				50 cubes			0.964		2–4.75	80	1642.28	0.485	12.42	9.94			6.8	
				50 cubes			0.964		2–4.75	90	1544.72	0.485	9.73	7.78			4.36	
				50 cubes			0.964		2–4.75	100	1447.15	0.485	7.61	6.09			3.65	

**Table 8:** Summary of statistical performance metrics.

Metric	Equation
R^2^	${\left(\frac{\sum \left({exp}_{i}-{\bar{exp}}_{i}\right)\left({mod}_{i}-{\bar{mod}}_{i}\right)}{\sqrt{\sum {\left({exp}_{i}-{\bar{exp}}_{i}\right)}^{2}\sum {\left({mod}_{i}-{\bar{mod}}_{i}\right)}^{2}}}\right)}^{2}$
MABE	$\frac{1}{n}\sum_{i=1}^{n}\left|{mod}_{i}-{exp}_{i}\right|$
MAPE	$\frac{1}{n}\sum_{i=1}^{n}\left|\frac{{exp}_{i}-{mod}_{i}}{{exp}_{i}}\right|\times 100$

**Table 9:** Formulated compressive strength equations and the associated models.

Type of Data	Proposed Model	Data Point	R^2^	MABE	MAPE
Experimental Data of This Study	$f_{chdpe}^{\prime }=\left(f_{c}^{\prime }\right)\left(e^{-0.0139(hdpe\%)}\right)$	3	0.999	0.095	0.425
Concrete Data	$f_{chdpe}^{\prime }=\left(f_{c}^{\prime }\right)\left(e^{-0.0212(hdpe\%)}\right)$	22	0.749	3.439	18.579
Mortar Data	$f_{chdpe}^{\prime }=\left(f_{c}^{\prime }\right)\left(e^{-0.0157(hdpe\%)}\right)$	19	0.975	1.716	8.799
All Data	$f_{chdpe}^{\prime }=\left(f_{c}^{\prime }\right)\left(e^{-0.0170(hdpe\%)}\right)$	41	0.882	2.542	14.970

**Table 10:** Performance assessment of the proposed compressive strength models.

Year	Reference	Data Point	R^2^	MABE	MAPE
2018	Badache et al. [19]				
	$f_{chdpe}^{\prime }=0.041\gamma_{chdpe}-44.498$	25	0.706	16.293	105.684
2021	Aocharoen and Chotickai [20]				
	$f_{chdpe}^{\prime }=0.0018(hdpe\%{)}^{2}-0.5644(hdpe\%)+45.3$	41	0.574	10.212	58.354
2023	Aocharoen and Chotickai [16]				
	$f_{chdpe}^{\prime }=\left(f_{c}^{\prime }\right)\left(1-0.009026(hdpe\%)\right)$	41	0.851	2.576	16.267
2025	Sancak and Ozyurt (this study)				
	$f_{chdpe}^{\prime }=\left(f_{c}^{\prime }\right)\left(e^{-0.0170(hdpe\%)}\right)$	41	0.882	2.542	14.970

**Table 11:** Equations and predictive models linking tensile strength to HDPE content.

Type of Data	Proposed Model	Data Point	R^2^	MABE	MAPE
Experimental Data of This Study	$f_{thdpe}^{\prime }=\left(f_{t}^{\prime }\right)\left(e^{-0.0084(hdpe\%)}\right)$	3	0.997	0.010	0.455
Concrete and All Data	$f_{thdpe}^{\prime }=\left(f_{t}^{\prime }\right)\left(e^{-0.0130(hdpe\%)}\right)$	15	0.886	0.175	6.149

**Table 12:** Equations and predictive models linking tensile strength to compressive strength.

Type of Data	Proposed Model	Data Point	R^2^	MABE	MAPE
Experimental Data of Punitha et al. 2021 [17]	$f_{thdpe}^{\prime }=1.1666{\left(f_{chdpe}^{\prime }\right)}^{0.2474}$	6	0.037	0.265	9.539
Experimental Data of Liu et al. 2022 [18]	$f_{thdpe}^{\prime }=0.2311{\left(f_{chdpe}^{\prime }\right)}^{0.8995}$	6	0.928	0.050	1.540
Experimental Data of This Study	$f_{thdpe}^{\prime }=0.3262{\left(f_{chdpe}^{\prime }\right)}^{0.6144}$	3	0.999	0.004	0.190

**Table 13:** Equations and predictive models linking flexural strength to HDPE content.

Type of Data	Proposed Model	Data Point	R^2^	MABE	MAPE
Experimental Data of This Study	$f_{rhdpe}^{\prime }=\left(f_{r}^{\prime }\right)\left(e^{-0.0077(hdpe\%)}\right)$	3	0.997	0.011	0.377
Concrete Data	$f_{rhdpe}^{\prime }=\left(f_{r}^{\prime }\right)\left(e^{-0.0159(hdpe\%)}\right)$	9	0.592	0.362	13.382
Mortar Data	$f_{rhdpe}^{\prime }=\left(f_{r}^{\prime }\right)\left(e^{-0.0083(hdpe\%)}\right)$	4	0.790	0.181	6.455
All Data	$f_{rhdpe}^{\prime }=\left(f_{r}^{\prime }\right)\left(e^{-0.0110(hdpe\%)}\right)$	13	0.512	0.390	14.836

**Table 14:** Equations and predictive models linking flexural strength to compressive strength.

Type of Data	Proposed Model	Data Point	R^2^	MABE	MAPE
Experimental Data of This Study	$f_{rhdpe}^{\prime }=0.5514{\left(f_{chdpe}^{\prime }\right)}^{0.5497}$	3	0.999	0.005	0.165
Concrete Data	$f_{rhdpe}^{\prime }=1.3367{\left(f_{chdpe}^{\prime }\right)}^{0.2252}$	9	0.065	0.279	10.509
Mortar Data	$f_{rhdpe}^{\prime }=0.7921{\left(f_{chdpe}^{\prime }\right)}^{0.4155}$	4	0.815	0.114	4.239
All Data	$f_{rhdpe}^{\prime }=0.9863{\left(f_{chdpe}^{\prime }\right)}^{0.3301}$	13	0.246	0.256	9.544

**Table 15:** Equations and predictive models linking modulus of elasticity to HDPE content.

Type of Data	Proposed Model	Data Point	R^2^	MABE	MAPE
Experimental Data of This Study	$E_{chdpe}=\left(E_{c}\right)\left(e^{-0.0152(hdpe\%)}\right)$	3	0.905	0.810	3.801
Concrete Data	$E_{chdpe}=\left(E_{c}\right)\left(e^{-0.0192(hdpe\%)}\right)$	10	0.755	4.003	18.525
Mortar Data	$E_{chdpe}=\left(E_{c}\right)\left(e^{-0.0142(hdpe\%)}\right)$	19	0.776	2.007	17.335
All Data	$E_{chdpe}=\left(E_{c}\right)\left(e^{-0.0152(hdpe\%)}\right)$	29	0.844	2.701	19.058

**Table 16:** Predicted models and their performance in linking modulus of elasticity to HDPE content.

Year	Reference	Data Point	R^2^	MABE	MAPE
2021	Aocharoen and Chotickai [20]				
	$E_{chdpe}=-0.1311(hdpe\%)+16.332$	29	0.440	5.474	23.457
2023	Aocharoen and Chotickai [16]				
	$E_{chdpe}=\left(E_{c}\right)\left(1-0.005522(hdpe\%)\right)$	29	0.622	5.506	45.126
2025	Sancak and Ozyurt (this study)				
	$E_{chdpe}=\left(E_{c}\right)\left(e^{-0.0152(hdpe\%)}\right)$	29	0.844	2.701	19.058

**Table 17:** Equations and predictive models linking modulus of elasticity to compressive strength.

Type of Data	Proposed Model	Data Point	R^2^	MABE	MAPE
Experimental Data of This Study	$E_{chdpe}=1.6015{\left(f_{chdpe}^{\prime }\right)}^{0.8741}$	3	0.915	0.696	3.147
Concrete Data	$E_{chdpe}=7.7388{\left(f_{chdpe}^{\prime }\right)}^{0.407}$	10	0.283	4.736	17.762
Mortar Data	$E_{chdpe}=0.7356{\left(f_{chdpe}^{\prime }\right)}^{0.9058}$	19	0.748	1.454	10.261
All Data	$E_{chdpe}=2.7369{\left(f_{chdpe}^{\prime }\right)}^{0.5655}$	29	0.150	6.583	42.696

**Table 18:** Predicted models and their performance in linking modulus of elasticity to compressive strength.

Year	Reference	Data Point	R^2^	MABE	MAPE
2018	Badache et al. [19]				
	$E_{chdpe}=\frac{f_{chdpe}^{\prime }-14.899}{0.08648}$	29	0.144	88.345	698.706
2021	Aocharoen and Chotickai [20]				
	$E_{chdpe}=0.3494(f_{chdpe}^{\prime })+2.2255$	29	0.144	7.218	52.783
2025	Sancak and Ozyurt (this study)				
	$E_{chdpe}=2.7369{\left(f_{chdpe}^{\prime }\right)}^{0.5655}$	29	0.150	6.583	42.696

## Data Availability

The original contributions presented in this study are included in the article. Further inquiries can be directed to the corresponding author.
